# Latest Advances in Biomimetic Cell Membrane-Coated and Membrane-Derived Nanovectors for Biomedical Applications

**DOI:** 10.3390/nano12091543

**Published:** 2022-05-02

**Authors:** Riccardo Rampado, Paolo Caliceti, Marco Agostini

**Affiliations:** 1 Department of Pharmaceutical and Pharmacological Sciences, University of Padua, Via Marzolo, 5, 35131 Padua, Italy; riccardo.rampado.farmacia@gmail.com (R.R.); paolo.caliceti@unipd.it (P.C.); 2 Nano-Inspired Biomedicine Lab, Insitute of Pediatric Research-Città della Speranza, Corso Stati Uniti 4, 35127 Padua, Italy; 3 General Surgery 3, Department of Surgical, Oncological and Gastroenterological Sciences, University of Padua, Via Nicolò Giustiniani 2, 35128 Padua, Italy

**Keywords:** biomimetic, nanoparticle, nanomedicine, membrane, coating, drug delivery systems

## Abstract

In the last decades, many nanovectors were developed for different diagnostic or therapeutic purposes. However, most nanosystems have been designed using a “bottom-up” approach, in which the basic components of the nanovector become assembled to achieve complex and specific behaviors. Despite the fine control of formulative conditions, the complexity of these systems often results cumbersome and difficult to scale-up. Recently, biomimetic materials emerged as a complementary or alternative design approach through a “top-down strategy”, using cell-derived materials as building blocks to formulate innovative nanovectors. The use of cell membranes as nanoparticle coatings endows nanomaterials with the biological identity and some of the functions of the cells they are derived from. In this review, we discuss some of the latest examples of membrane coated and membrane-derived biomimetic nanomaterials and underline the common general functions offered by the biomaterials used. From these examples, we suggest a systematic classification of these biomimetic materials based on their biological sources and formulation techniques, with their respective advantages and disadvantages, and summarize the current technologies used for membranes isolation and integration on nanovectors. We also discuss some current technical limitations and hint to future direction of the improvement for biomimetics.

## 1. Introduction

In the last years, nanomaterials have been mainly formulated according to a “bottom-up” approach, in which the single chemical components are assembled or even chemically synthesized to obtain the final nanovector with the desired features [1]. This approach led to important scientific milestones and the approval of nanoformulations for the treatment of many diseases, consolidating nanotechnology as an innovative and fruitful field of inquiry [2]. The need to improve synthetic nanoparticles (NPs) tissue specificity spawned formulations with disparate features such as pH-dependent [3,4], temperature-dependent, redox-dependent [5], and enzyme-responsive [6] materials with exponentially increasing complexity. However, this quest for nanomaterials with increasingly specific behaviors and higher targeting efficiencies led to very complex nanoplatforms, which presented limited applications in vitro and even less success when tested in vivo [7], and require cumbersome chemical synthesis. This created the artificial hurdle of reproducing their complexity on a large scale for industrial production, hindering their clinical translation. Finally, novel synthetic materials for biomedical applications always pose issues of biocompatibility.

On the other side of the spectrum of innovative treatments are biological therapies, which include many different strategies, ranging from monoclonal antibodies [8], to viral vectors [9], extracellular vesicles [10], and cell therapy [11]. These therapies proved to be game changers, providing new therapeutic options, and opening the way to entirely new ways to think about the treatment of many pathologies. However, the complexity of biological therapies and their production steps pose some issues such as their reproducibility on a large scale, safety concerns over some of these treatments (e.g., viral vectors and cellular therapies), and difficulty in complete characterization [12,13].

**Figure 1:**
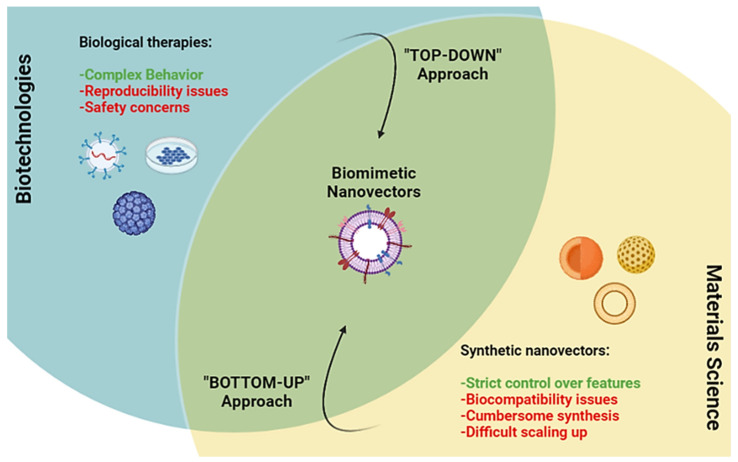
Schematic representation of how biomimetic nanovectors fit at the crossroads of biological therapies and synthetic nanovectors. For each field, its relative advantages are presented in green and disadvantages in red. This image was created using Biorender.com (accessed on 1 March 2022).

Thus, in the last years, the concern about “over-engineered” synthetic nanomaterials led to a renewed interest in using formulations with simpler designs, focusing on biocompatibility and scalability of production. At the same time, the quest to streamline the production of biological therapies led to a “top-down” approach, selecting the essential components of biologicals necessary to achieve the desired effect as building blocks. The rise of biomimetic nanomaterials attempts to address this call to reductionist innovation as an intersection between traditional synthetic nanomedicine and biotechnologies, offering the best of both words in terms of biocompatibility, safety, and scalability of formulations with complex behaviors (Figure 1).

Among the different biomimetic approaches investigated to improve nanoparticles (NPs) pharmacokinetics, cell membranes and their components have emerged as a fascinating opportunity [14]. The plasma membrane and the markers expressed on it separate the inside of cells from their environment. The plasma membrane defines the biological identity and behavior of cells and has many essential functions, including mediating cell interactions with soluble molecules that work as biological signals; their interactions with other cells, including their recognition as “self” by the immune system, and the homing ability of circulating cells to specific tissues via adhesion molecules. Thus, recapitulating the membrane features on the surface of nanomaterials can also modulate their behavior, depending on the cells the membranes or membrane markers are derived from.

In this review, we discuss the latest advances in the formulation of biomimetic nanovectors using cell membranes and membrane components to improve the features of synthetic nanomaterials, or as standalone drug delivery systems. Furthermore, in addition to presenting the most notable advances in the field, we also try to offer a systematic classification of biomimetic nanomaterials based on their cell source and formulation technology. From this bedrock, we extract the main general applications biomimetics defining three main functional classes. In the final section, we also discuss some current limitations that remain to be completely addressed as well as new possible developments in the field. This systematic approach would offer to the reader a more unified view on the field of biomimetics, constituting a potential toolbox for the design of future cell membrane based nanovectors.

### Overview of the Classes, Biological Sources, and Functions of Membrane Coated and Membrane Derived Biomimetic Nanovectros

**Figure 2:**
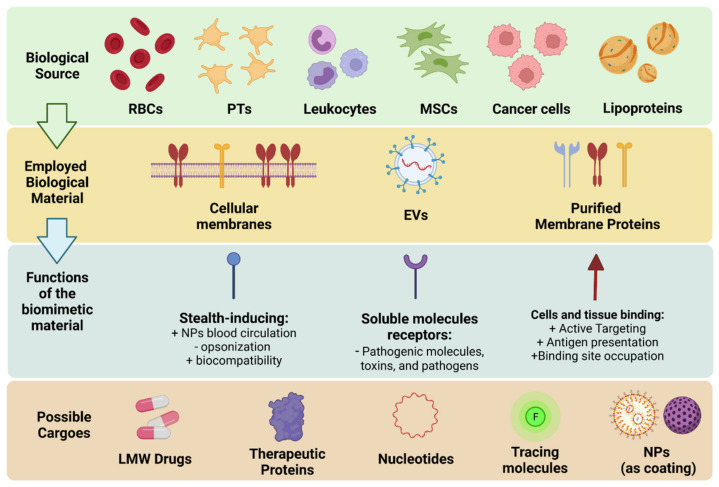
Schematic representation of the current biomimetic approach toolbox in term of source cells, biomimetic strategies, membrane proteins functions, and cargoes. This image was created with Biorender.com (accessed on 1 March 2022). EVs: extracellular vesicles; LMW: low molecular weight; MSCs: mesenchymal stem cells; NPs: nanoparticles; PTs: platelets; RBCs: red blood cells.

The use of biomimetic nanomaterials offers the unprecedented opportunity to employ highly biocompatible materials with a wide range of complex behaviors that can be adapted to many pathologies, including the ability to avoid immune clearance, deliver the cargo through specific surface receptors, bypass biological barriers and absorb toxic molecules [15]. Figure 2 summarizes the cells biomimetic materials are most commonly derived from, the materials that can be extracted, and the main functions exerted by the proteins employed.

The first of function is mediated by membrane proteins which are ligands for specific molecules expressed by tissues, providing active targeting. This group of proteins includes integrins and adhesion molecules expressed by immune cells or platelets (PTs). It is important to note that these molecules can exert their biologic functions upon binding to their target, triggering intracellular responses, resulting in bioactivity on their own. Furthermore, these molecules can work as antagonists by competing for their binding sites with autologous ligands. This class also includes antigens that are recognized by immune cells and are used to formulate nanovaccines.

The second class is constituted by proteins that make the biomimetics recognized as “self-entities” by the immune system, normally by inhibiting complement activation or avoiding immune clearance by phagocytosis during circulation or residence into tissues. This class includes molecules such as CD47 expressed by macrophages, red blood cells (RBCs), PTs, and even some tumor cells that can bind to the SIRPα onto myeloid immune cells inhibiting phagocytosis [16]. This results in a prolonged plasmatic half-life of the nanovectors, offering them more time to reach the target tissue. The CD47-SIRPα interaction is perhaps the immune-avoidance mechanism most discussed when designing and testing new biomimetic formulations from different cell lines.

The third group of proteins instead includes proteins that are receptors for soluble molecules, and they can bind specifically to them, inhibiting their function and working as “decoys”. This class includes all receptors for chemokines, interleukins, and growth factors [17].

**Figure 3:**
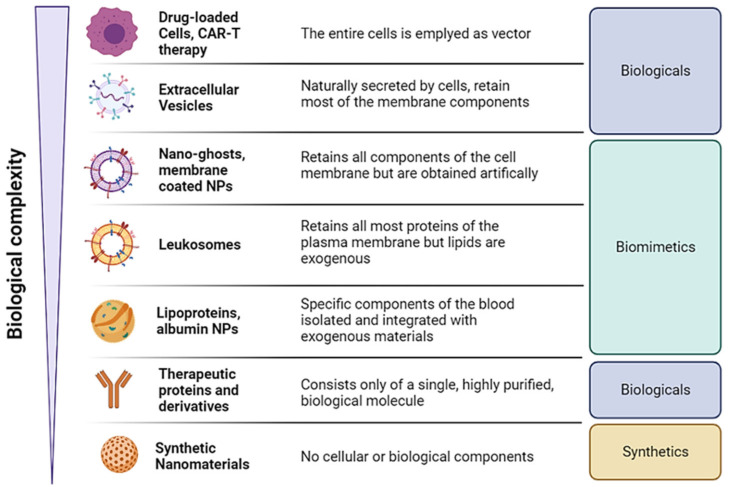
Schematic representation of biological therapies classified based on their resemblance to actual cells. This figure was produced using Biorender.com (accessed on 1 March 2022).

It also possible to classify biomimetic materials based on their resemblance to the complexity of cells (Figure 3). Specifically, stating from cell therapies, extracellular vesicles (EVs) can be considered as cells fragments that recapitulate part of the cells surface markers and can carry cells cargo (e.g., cytosolic proteins, RNAs). Since EVs are naturally secreted by all cells, they still belong to biological therapies and discussion on their uses goes beyond the scope of this work. We direct the reader to ad hoc reviews on the matter [18,19]. At the bottom of the spectrum, there are instead synthetic materials with very limited to no resemblance to cells, and biologics composed of highly purified proteins (e.g., soluble receptors and monoclonal antibodies) [20]. Biomimetic nanovectors position themselves between these two classes of therapeutic approaches, since they are derived from cells by extracting a specific set of materials. These materials can range from complete cell membranes to only membrane proteins which then become reconstituted as nanovectors, and even non-cellular biomaterials such as lipoproteins. In the next pages, we will present recent examples of different membrane-coated or membrane-based nanovectors for theranostic applications based on their formulative technologies and cellular origins.

## 2. Membrane Coated and Membrane-Based Nanomaterials

The most common approach to formulate biomimetic nanomaterials is the use of whole cellular membranes as a coating for NPs. This approach aims to translate on nanoparticles the entirety of the plasma membrane [21]. This is achieved by isolating the cellular membranes through the hypotonic treatment, sonication, homogenization, or a combination thereof of the source cells to isolate cell membranes, and their subsequent sonication or extrusion to reduce the membranes’ size to the nano range.

Among the many cell types that can be used for particle coating, the most popular are certainly RBCs, PTs [22], and leukocytes [23] (Figure 2). This is not surprising considering that most NPs are designed for intravenous administration. Therefore, cells that are present in the systemic circulation are prime candidates for this endeavor since they would not be considered as “out of place” by the organism immune system.

RBC membranes present on their surface proteins, such as CD47 that can bind receptors on the leukocyte membrane (i.e., SIRPα), inhibiting their clearance and providing the coated NPs with much longer plasmatic half-life. Similar receptors are also present on the surface of PTs, leukocytes, and even in some instances cancer cells. Another advantage of RBCs and PTs is their lack of nuclei and most intracellular organelles, making them natural “cells ghosts” which can be readily used to coat NPs. RBCs and PTs are also abundant and easy to isolate from the blood, enabling the use of a patient’s own cells for particle coating, further increasing the biocompatibility of nanomaterials. PTs express specific adhesion molecules that enable PTs binding onto the surface of damaged endothelia, such as in local inflammations, tissue damage, and even some tumors [24].

Leukocytes can also naturally home to inflamed endothelia expressing adhesion molecules such as PECAM-1, VECAM-1, ICAM-1, and ICAM-2 [25] to target inflamed tissues, similarly to PTs. Leukocytes present on their surface also an array of cytokines receptors that can work as scavengers for circulating inflammatory molecules, removing them from the organism. Furthermore, immune cells also express many cytotoxic receptors (e.g., FasL, PD-1) [26,27], and co-stimulatory proteins (e.g., MHC-I/II) [28] that can be used to either induce the apoptosis of target cells via direct interaction or elicit the adaptive immune response as natural adjuvants, respectively.

Other options for NPs coating are mesenchymal stem cells (MSCs) membranes, which have gained attention for their ability to selectively accumulate into tumors [29].

Tumor cells have also been used thanks to their peculiar features to formulate biomimetic nanovectors. The gene expression dysregulation and the mutations accumulated by tumor cells often result in the expression of neo antigens that could be leveraged to obtain novel anticancer nanovaccines to induce an antitumor immune response though the presentation of tumor neoantigens [30]. Another possible application of tumor cell membranes stems from the natural tropism that tumor-derived extracellular vesicles display to their tissue of origin. Thus, coating nanomaterials would yield membrane coated nanovectors that could target the tumor cells they are derived from after injection [31].

### 2.1. RBC-Coated NPs

RBC coating has been used for traditional drug delivery applications, specifically as a coating to improve the biocompatibility and pharmacokinetics of synthetic nanomaterials. This approach was pioneered by Hu et al. who coated Poly-lactic-co-glycolic acid (PLGA) NPs using murine RBCs membranes [32]. This formulation demonstrated good membrane proteins translocation onto the NPs, remarkable colloidal stability under storage and in FBS, as well as an almost doubled plasmatic half-life after intravenous injection in mice compared with similar polymeric particles coated with poly-ethylene-glycol (PEG) as a stealth inducing agent. This study indeed demonstrated the high potential of RBC membrane coating of synthetic nanomaterials as a simple and efficient way to lower their clearance.

A similar use of RBC membrane is their use to coat PLGA NPs loaded with the Hedgehog inhibitor cyclopamine to treat pancreatic ductal adenocarcinoma [33], which resulted in prolonged drug half-life and improved drug accumulation into the tumor by three times, reducing tumor growth by 80% in a xenograft pancreatic adenocarcinoma murine model.

A different example of RBCs membrane application is offered by Zhou et al. [34]. In this study, Doxorubicin (DOXO) loaded, pH-sensitive dextran NPs are coated with RBC membrane and are functionalized with the active targeting moiety Angiopep-2, which can bind to low density lipoproteins-related receptors expressed by the blood–brain barrier (BBB) vessels and by glioblastoma cells. These particles were uptaken at a fast rate by glioblastoma U87MG cells with remarkable cytotoxic effect. Furthermore, they prolonged the DOXO half-life in vivo by 10 times compared to uncoated particles and accumulated with high efficiency in the tumor in in vivo orthotropic murine glioblastoma models, crossing the BBB and prolonging animal survival compared to the free drug (more than 20 days more).

Fu et al. [35] instead focused on the development of vincristine-loaded solid lipid NPs (SLNs) coated with RBC membrane functionalized with the T7 and NGR peptides, to target the transferrin receptor (TfR) expressed on the BBB, and the tumor-expressed CD13 marker respectively, as a drug delivery vector against glioma. These particles efficiently targeted the brain in zebrafish and mice orthotropic models, and reduced tumor growth by 50%, almost doubling the animals’ survival time.

Another work by Liu et al. [36] investigated ROS-sensitive arylboronic ester-based biomimetic nanocarriers loaded with the photosensitizer chlorine e6 and the hypoxia-activated antitumor prodrug tirapazamine to target tumor hypoxia and enable combined photodynamic therapy (PDT) and chemotherapy. The RBCs coating enabled the colloidal stabilization of NPs and increased the uptake by tumor cells twofold, especially when functionalized with the RGD peptide, which binds αV integrins and neuropilin-1 receptors overexpressed in some breast cancer cells. These membrane-coated nanovectors very efficiently targeted tumor tissues (with comparable levels to the liver) in breast tumor bearing mice and upon near infrared light (NIR) irradiation reduced tumor growth and weight by over 90%.

However, RBC membrane coating does not exempt NPs from other important considerations in their design, including their shape or size, which can still significantly influence their in vivo fate. An example of this is offered by a recent work by Li et al. [37] demonstrated how smaller, spherical RBCs membrane coated PLGA particles (80 nm) had a longer half-life (30 h) and reduced liver accumulation compared to bigger particles (100 and 200 nm particles, with an half-life around 10 h). This could be due to reduced liver filtration via sinusoid capillaries. Thus, smaller particles appeared to be more suitable to enable long circulation time.

Another study applied hyaluronidase-sensitive, paclitaxel and pheophorbide A-loaded particles coated with RBCs membranes to treat breast cancer, which presented on their surface a PD-L1 binding peptide as synergistic molecules for immunotherapy [38]. These particles confirmed the size trends exposed before, with bigger particles being cleared faster in vivo. These nanovectors accumulated into the tumor tissue of breast cancer-bearing mice, inducing immunogenic cell death, the local accumulation of CD4+ and CD8+ T-cells in the tumor stroma, and hindering tumor growth by over 90% compared to the untreated control and two times more than the untargeted NPs.

Another iteration of this approach is presented by Zhang et al. [39], which formulated RBC membrane-coated PLGA particles loaded with gambogic acid and functionalized with anti-epidermal growth factor (EGFR) iRGD peptide for colorectal cancer (CRC) active targeting. These particles showed remarkable colloidal stability and very slow drug release, suitable for long circulation, and at the same time efficient tumor cells targeting in vivo, reducing CRC growth by almost 90% and improving animals’ survival by 60%.

RBCs membrane coating has been used to complement radiotherapy by improving the pharmacokinetics of radioisotopes. This was tested by Lee et al. [40] to deliver the radioisotope Zr-89 for the imaging of CRC. This formulation demonstrated doubled half-life compared to bare NPs, and good tumor targeting in CRC cells bearing mice, enabling efficient positron emission imaging.

In a study from Meng et al. [41], iron oxide NPs were coated with RBC membranes and functionalized with antibodies against circulating prostate cancer cells to remove them magnetically. RBC membranes provided colloidal stability to NPs, reducing the absorption of plasma proteins onto their surface correlated to reduced NPs plasmatic half-life). These particles successfully isolated tumor cells from prostate cancer patients’ samples with over 95% efficiency, demonstrating the synergistic activity of RBC coating and antibody labelling for potential diagnostic applications.

Liang Fang Zhang was among the first to use RBC coating as a nanosponge against bacterial infections. A remarkable example of this concept is offered in a recent work [42], in which PLGA particles were coated with RBC membranes. These NPs demonstrated the ability to greatly reduce the hemolytic abilities of *Staphylococcus aureus* toxins both in vitro (40%) and in vivo, resulting in a dose-dependent increase in survival in animal models of systemic infection, reducing both the toxins damage to lungs and systemic inflammation markers (i.e., Nf-KB expression, lung edema, and alveolar thickness). Notably, this approach resulted useful even in treating drug resistant bacteria.

**Figure 4:**
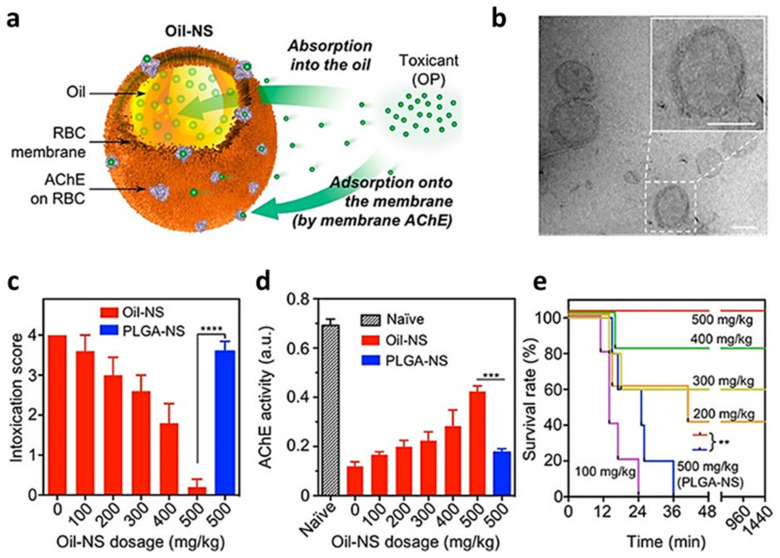
(**a**) Schematic representation of Oil nanospheres (Oli-NS), composed of a small oil droplet stabilized by red blood cells (RBC) derived membrane as detoxifying agent for organophosphate (OP). (**b**) Representative transmission electron microscope image of the spherical core–shell structure of Oil nanospheres. Scale bar = 100 nm. In vivo efficacy of Oil nanospheres against organophosphates intoxication. Mice were first injected intraperitoneally with oil or PLGA nanoparticles at different doses They were then challenged 2 min later by a single subcutaneous injection of organophosphates at 0.7 mg/kg. (**c**) Intoxication signs of mice were scored at 10 min post-injection. (**d**) Acetylcholinesterase (AChE) activity of blood measured at 10 min post-POX injection. (**e**) Survival rates of mice over 24 h after POX injection. In all studies, n = 5 per group. (** *p* < 0.01, *** *p* < 0.001, and **** *p* < 0.0001). Figure adapted with permission from *ACS Nano* **2019**, *13*, 7209–7215 (https://pubs.acs.org/doi/10.1021/acsnano.9b02773). Copyright 2019 American Chemical Society (accessed on 1 March 2022).

This system was improved using a liquid oil core instead of PLGA NPs as core (Oil-NS) (Figure 4). The use of a liquid core makes the nanosponges able to strongly bind hydrophobic toxins through their receptors on the RBCs membranes, which then get partitioned into the core itself. This results in a sink condition in which the core works as a functional compartment in the PLGA formulation. This intuition was demonstrated by Chen et al. [43], who used this platform to reduce the toxicity of different acetyl cholinesterase toxins. Oil-NS demonstrated good binding activity and toxins removal in vitro (Figure 4c,d), translating in the efficient systemic detoxification in vivo, rescuing by 40% acetyl cholinesterase activity and resulting in complete animal survival at 500 mg/kg of dose (Figure 4e).

However, the cell membrane coating of particles presents some technical challenges. Specifically, the process of membrane coating occurs randomly, and does not follow the important in–out orientation of the cellular membrane. This could result in the wrong orientation of proteins on the surface of the particles, leading to the outer exposure of otherwise intracellular protein domain, which are not functional towards the NPs environment. This concern was addressed by Xie et al. [44], who functionalized cationic liposomes with a peptide ligand to bind the intracellular domain of Band 3, an important protein present onto RBCs necessary for immune escape. By coating these liposomes with RBC membranes, the authors ensured the correct orientation of band 3 protein. This system resulted in RBCs coated liposomes with long stability and doubled the plasmatic half-life of PEGylated NPs. Furthermore, the particles could efficiently target the infected tissues of *Candida albicans* bearing mice, and efficiently absorbed the fungal toxins, increasing animals’ survival compared to untreated and even PEGylated NPs.

Luk et al. [45] have also used RBCs coated NPs as a decoy for autoimmune hemolysis, a pathology in which the immune system produces antibodies against its own RBCs, causing complement-mediated and immune-mediated hemolysis. RBC membrane-coated PLGA NPs efficiently bound to anti-RBCs sensitized B-cells in vitro, and selectively targeted this cell population in a murine model of autoimmune hemolysis. This could represent a future approach for drug delivery in autoimmune pathologies.

RBC coating has also been applied to regenerative medicine. Liang et al. [46] encapsulated growth factors derived from MSCs medium into PLGA NPs and coated them with RBCs membranes to induce liver regeneration after acute hepatic failure. This platform demonstrated remarkable colloidal stability, slow release of the loaded factors, and the ability to promote hepatocytes activity in vitro. This translated in a marked decrease in liver enzymes in the blood (i.e., ALT and AST, by almost 95% after 7 days) and of inflammatory markers (IL-6, IL-1beta, and TNF-α, 20% compared to simple conditioned medium) in murine hepatic carbon chloride induced failure, with significant improvement in animal survival.

### 2.2. PT-Coated NPs

Wang et al. [47] offered an example of PT membranes used as NPs coating. In this study, bufalin-loaded PLGA NPs formulated via nanoprecipitation were coated with PT membranes derived from blood to provide them with long circulation and tumor homing abilities through the P-selectin surface protein interaction with CD44 expressing hepatocarcinoma cells. Their uptake was indeed mediated by P-selectin since non-coated particles were not uptaken since anti-selectin treatment could reduce their uptake by blocking the targeted receptors. When used in vivo, these particles did not show any toxic effect while accumulating in the tumor tissue in an ectopic murine hepatocarcinoma model and delivering the antitumor molecules bufalin, reducing tumor volume and weight by 80%.

A similar type of poly-lactic-acid (PLA) particles [48], loaded with the Toll-like receptor-8 activator resiquimod successfully delivered their cargo to different CRC and breast cancer cell lines in vitro. PTs coated particles demonstrated increased uptake by tumor cells in a murine model of CRC. These particles accumulated in CRC bearing mice and showed prolonged retention when injected intratumorally (half-life around 24 h compared to few hours of PEGylated NPs). Furthermore, they were able to elicit immune cells activation by increasing the expression of MHC-II by myeloid and dendritic cell markers in the draining lymph nodes of tumor bearing mice, as well as a higher presence of CD4+ memory T cells. Harvested tumor sections also showed an increased infiltration of CD4+ and CD8+ T cells in the tumor stroma. These particles ultimately eradicated the tumor mass in vivo and hindered tumor volume in a breast cancer mouse model by 90%, preventing the formation of metastatic noduli.

**Figure 5:**
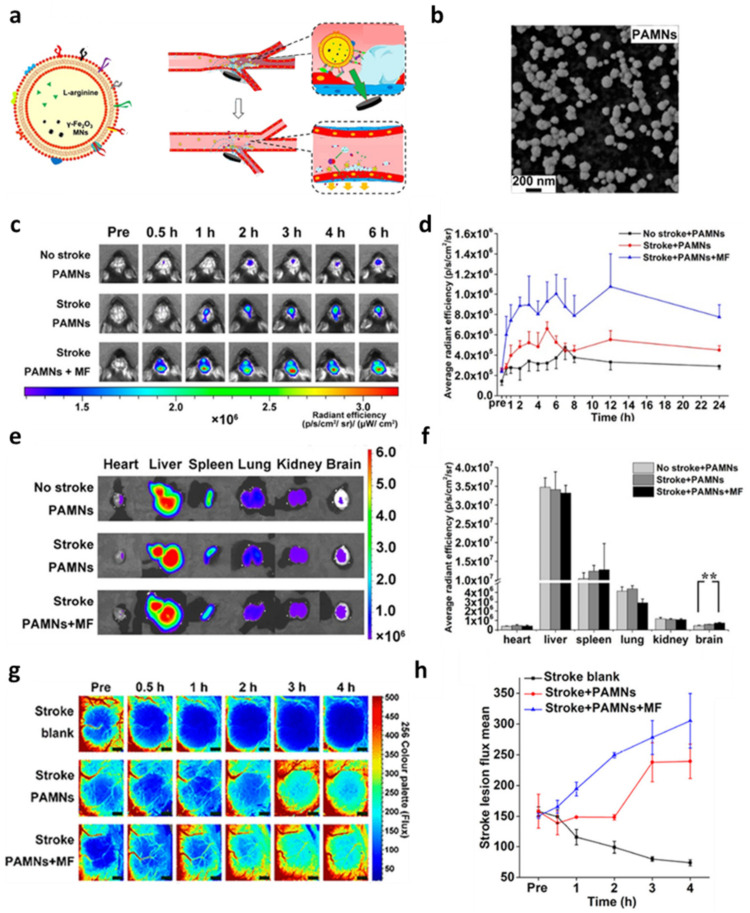
(**a**) Schematic representation of platelet membrane enclosed L-arginine and magnetite nanoparticles (PAMN) structure and in vivo targeting moieties. As PT membrane-coated biomimetic nanovector, PAMNs recapitulate the natural features of the PT membranes that expose on their surface specific binding proteins, providing active targeting to damaged vessels and immune escape. Through the mimetic properties of PT membranes and the application of a magnetic field (MF), the PAMNs reach the stroke lesion more quickly to achieve rapid targeted delivery of L-arginine. The in situ generation of nitric oxide (NO) induces vasodilation and reduces PLT aggregation. (**b**) Scanning electron microscopy characterization showing the surface structure of PAMNs. (**c**) NIR images of mice before and after injection with labeled PAMNs over time and their relative quantification (**d**). (**e**) Ex vivo NIR imaging of excised major organs 6 h after PAMN injection and its relative quantification (**: *p* < 0.01) (**f**). (**g**) Color-coded images showing blood reperfusion in the ischemic lesion within 4 h after thrombus formation that is comparable to the recognized therapeutic time window (4.5 h). Scale bar: 1 mm and their relative quantification (**h**). Figure adapted with permission from *ACS Nano* **2020**, *14*, 2024–2035 (https://pubs.acs.org/doi/abs/10.1021/acsnano.9b08587). Copyright 2019 American Chemical Society (accessed on 1 March 2022).

The natural tropism of PTs to damaged and inflamed endothelia prompted the use of PT membranes to target vascular diseases. In a study from Li et al. [49], magnetite NPs and L-arginine were loaded into PT membranes (PAMNs) to achieve efficient targeting of thrombi after stroke, with the aim to induce nitrous oxide production through L-arginine as substrate, in turn causing blood vessels dilation, and reducing platelets aggregation on the thrombi (Figure 5a). Magnetite NPs can accumulate into the affected site by applying an external magnetic field (PAMN + MF). PAMNs + MF efficiently accumulated into central nervous system (CNS) blood vessels in vivo in murine stroke models (Figure 5c–f), reducing by over 60% platelets local aggregation and partially restoring blood flow into the affected tissue (Figure 5g,h).

Wang et al. [50] created poly-amino-amide dendrimer-based nanoclusters loaded with the endothelia protective agent JQ1 and coated with PT membranes for the targeting and treatment of arterial stenosis, as an alternative to drug loaded highly invasive stents. This innovative approach, upon intravenous injection, efficiently targeted only the affected sites as the actual PTs (over five times more compared to uninjured controls), and remodulated gene expression in stenotic arteries of murine models, increasing the vessels’ lumen and decreasing the hyperplasia of the endothelia.

PTs membranes have also been used as a decoy mechanism to protect from toxic molecules. One example is offered by Kim et al. [51], who used PT-coated PLGA NPs as a dampening agent against *Staphylococcus aureus* toxins. These particles prevented toxins from damaging circulating PTs and prevented toxic damage to macrophages and neutrophils. They also demonstrated the ability to boost PT activation, and macrophages and neutrophils oxidative stress, reinforcing their bactericidal activity in vitro and in vivo in a model of systemic *Staphylococcus aureus* infection. This resulted in halved expression of IL-6 and bacterial count, and greatly improved survival. This study evidences the potential ability of PTs to modulate the immune system as intrinsically active nanomaterials.

Another decoy-based use of PTs coating is their use to remove anti-platelets antibodies in immune thrombocytopenia. In this pathology, the body produces antibodies against its own PTs, resulting in their fast clearance and coagulation dysfunction. A recent work by Wei et al. [52] demonstrated how these coated PLGA particles bonded anti-platelets antibodies in vitro in a dose-dependent way. This resulted in remarkable detoxifying effects in a murine model of immune thrombocytopenia, in which the anti-PTs immunoglobulins titer was much decreased, rescuing the amount of PTs and the bleeding time to normal values.

### 2.3. Leukocytes-Coated NPs

Leukocyte-coated NPs can work as a molecular nano-sponge, binding to specific pathological molecules or viruses. An example of this it given by Wei et al. [53], in which PLGA NPs were coated with CD4 T-cells against HIV infection. These particles retained all the major membrane markers of T-cells including CD4, CCR5 and CXCR4 involved in HIV virus internalization. T-cells coated particles bonded HIV receptors in vitro very efficiently, reducing by over 80% the T-cells death caused by HIV virus itself.

**Figure 6:**
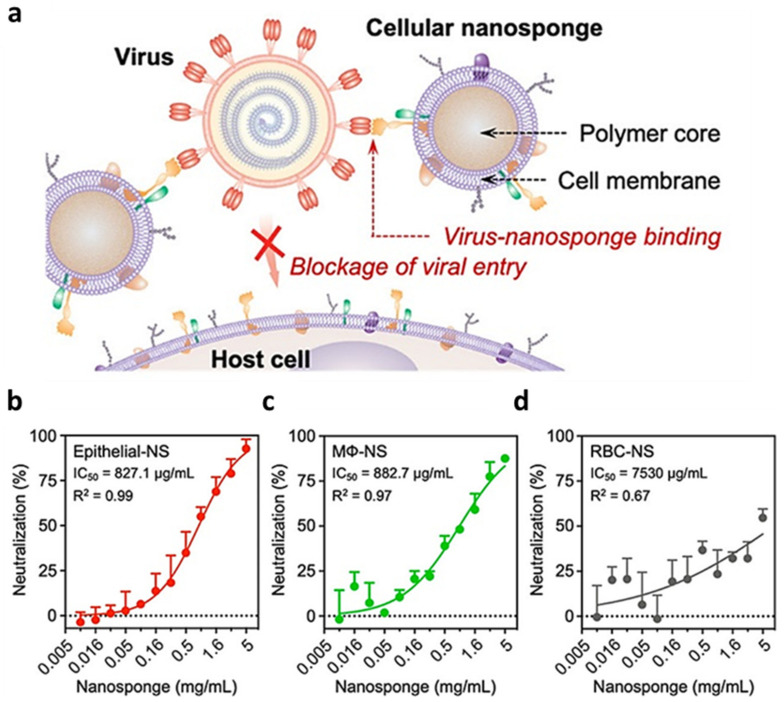
(**a**) Schematic representation of cellular nanosponges inhibiting SARS-CoV-2 infectivity. The nanosponges are formulated by wrapping polymeric nanoparticles with cell membranes from target cells such as lung epithelial cells and macrophages (MΦs). The resulting nanosponges (denoted “Epithelial-NS” and “MΦ-NS”, respectively) inherit the surface antigens of the source cells and serve as decoys to bind with SARS-CoV-2. To block viral entry and inhibit viral infectivity. (**b**) Epithelial-NS, (**c**) MΦ-NPs, and (**d**) nanosponges made from red blood cell membranes (control) was tested using live SARS-CoV-2 viruses on Vero E6 cells. In all data sets, n = 3. Data are presented as mean + standard deviation. Horizontal dashed lines mark the zero levels. Figure adapted with permission from *Nano Lett.* **2020**, *20*, 5570–5574 (https://pubs.acs.org/doi/10.1021/acs.nanolett.0c02278). Copyright 2019 American Chemical Society (accessed on 1 March 2022).

The robustness of nanosponges as decoys against infective agents was demonstrated in a recent work by Zhang et al. [54], who formulated PLGA NPs coated with lung epithelial cells (Epithelial-NS), macrophages membranes (MΦ-NS) as binding agents against the SARS-CoV-2 virus (Figure 6a). Both cell types present on their surface the receptors the ACE2 receptor used by the virus for cell adhesion. These NPs demonstrated remarkable biocompatibility and high affinity in binding viral particles in vitro in a dose-dependent trend (Figure 6b–d), consolidating this decoy strategy as versatile in many pathological contexts.

NPs can be used to deliver enzymes to treat metabolic diseases, re-establishing the homeostasis of specific substances. One example is hyperuricemia, caused by an excess of circulating uric acid, and its accumulation in body extremities as small crystals. The amount of circulating uric acid can be reduced by the administration of the uricase enzyme to convert uric acid in soluble allantoin. This protein, however, is quickly removed from circulation. To increase the half-life of the enzyme, Zhuang et al. [55] encapsulated it into metal organic frameworks NPs coated with RBC or macrophage membranes. Both coated formulations demonstrated good stability and biocompatibility, maintaining the enzyme conversion. Macrophage membranes also preserved several cytokines receptors (i.e., IL-1R, TNF-α receptor, and IL-6R), binding these inflammatory molecules which contribute to hyperuricemia-derived inflammation. Macrophage-coated particles demonstrated in vivo 30% improved therapeutic efficacy compared to RBC coated NPs thanks to the dual therapeutic activity of the enzyme and the decoy action of the particles themselves.

Antigen presenting cells (APCs) are naturally involved in antigen recognition and processing. Thus, they naturally present on their surface an array of co-stimulatory proteins that are necessary to activate effector cells (i.e., NK cells and T cells), ultimately inducing the immune response. Transposing these co-stimulatory proteins and the processed antigens onto NPs thus could result in natural nanovaccines able to induce an immune response against tumor neoantigen. This intuition was tested in a recent work by Chen et al. [56] who derived cancer cell membrane coated PLGA NPs as a starting platform to expose tumor antigens to dendritic cells (DCs) as primary APCs. DCs thus would process the antigens present them onto their membrane. By purifying the membranes of activated DCs and coating PLGA NPs, the authors obtained the final nanovaccines. These nanovaccines demonstrated good size and a core-shell structure, confirming the retention of antigen after NPs coating. The nanovaccines efficiently interacted with and activated mice derived T cells ex vivo, inducing their proliferation and expression of CD3, CD8, and IFN-γ. Nanovaccines were obtained both from OVA-expressing B16-OVA and from hepatic cancer Hepa 1-6 cells. After in vivo injection in the respective tumor bearing mice, both these treatments resulted in T cells activation both in the spleen and the tumor mass, compared to NPs coated with non-primed DCs. This resulted in the increased expression of markers for APCs such as MHC-II, CD80, and CD86, T cells markers (i.e., CD3, CD4, and CD8) and pro-inflammatory cytokines (IFN-γ and TNF-α). This resulted in almost complete inhibition of tumor growth. Furthermore, this response was tumor specific since OVA-derived nanovaccines did not show any benefit in Hepa 1-6 tumors and vice versa. Of note, the treatment also synergized with an anti PD-1 antibody used as immune checkpoint inhibitor, completely eradicating the Hepa 1-6 tumor mass. This remarkable work demonstrates the immune-modulatory potential of biomimetic nanomaterials.

A different study by Kang et al. [57] instead tested T cell membrane coated PLGA particles as intrinsically active anticancer nanovector and as drug delivery system for the antitumor drug dacarbazine. T cell membrane coating in this case has multiple functions: they offer adhesion molecules (e.g., LFA-1 and ICAM-1) to achieve active targeting to the inflamed tumor associated endothelia; they present PD-1 that can work as immune checkpoint inhibitors against tumor cells; they express FasL which can induce tumor cells apoptosis via direct interaction; and cytokines receptors such as TGF-β1 R can work as scavengers to remove local immune suppressive molecules. The presence of all these markers was confirmed on the surface of the coated NPs. Furthermore, these particles demonstrated the ability to induce melanoma B16F10 cancer cells apoptosis, T cells activation, and TGF-β1 binding in vitro. After in vivo injection in melanoma murine models, T cell coated particles efficiently reached the tumor mass and increased the immune infiltrate of CD8+ T cells, while reducing Treg cells. This resulted in the reduction of over 60% of the tumor mass. These remarkable results were confirmed also in melanoma metastasis and lung cancer, albeit in the latter case particles were loaded with dacarbazine.

A similar approach for tumor active targeting and PD-L1 inhibition was tested by Zhai et al. [58]. The authors in this case induced the expression pf PD-1 on T cell membranes via viral transfection and then used this enriched membrane to coat bovine serum albumin (BSA) core loaded with the IFN-inducer ORY-1001 to induce antitumor immune activation. These particles demonstrated the ability to induce IFNα and IFNβ expression in triple negative breast cancer (TNBC) 4T1 cells, as well as reducing tumor cells PD-L1 expression. After IV injection in vivo in murine models of TNBC, the membrane-coated nanovectors efficiently accumulated into the tumor compared to non-PD-1 enriched particles, with analogous favorable effects on the intratumor levels of IFNα, IFNβ, and PD-L1. Furthermore, the increased IFN resulted in higher tumor infiltration of T cells, DCs, higher secretion of proinflammatory molecules such as IL-6, TNF-α and granzymes, reduced local T_reg_ cells, and ultimately abolished tumor growth and improved animals’ survival.

### 2.4. Tumor Cell-Coated NPs

**Figure 7:**
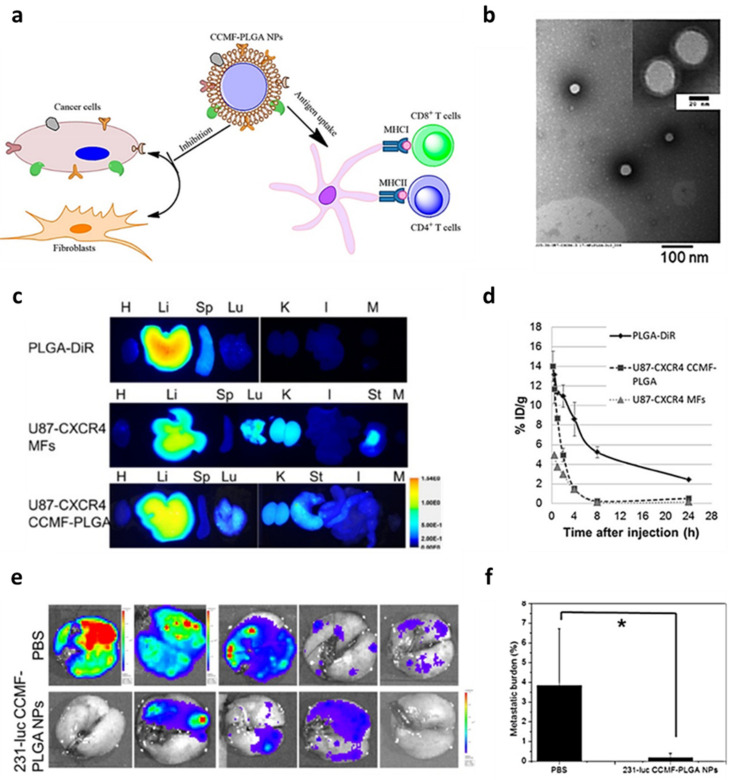
(**a**) Schematic illustration of the use of cancer cells membrane fraction-coated PLGA nanoparticles (CCMF-PLGA-NPs) to inhibit fibroblasts-cancer cells interactions and induce antitumor immunity via antigen presenting cells. (**b**) Representative TEM images of U87 cells-derived CCMF-PLGA-NPs (U87-CXCR4 CCMF-PLGA NPs) with insets showing high-magnification images. Scale bar is 20 nm. (**c**) Representative fluorescence images of major organs harvested at 24 h post injection of DiR-labelled PLGA particles (PLGA-DiR), U87 membrane fractions (U87-CXCR4-MFs), or U87-CXCR4 CCMF-PLGA NPs (100 μg for each). H, heart; Li, liver; Sp, spleen; M, muscle; Lu, lung; K, kidney; I, intestine; and St, stomach. (**d**) Pharmacokinetic curves of PLGA NPs, U87-CXCR4 MFs, and U87-CXCR4 CCMF-PLGA NPs in mouse plasma over a period of 24 h post injection of NPs (100 μg for each) through the tail vein. (**e**) Ex vivo bioluminescence images of metastatic nodules in lung after injection of 231-luciferase labelled CCMF-PLGA (231-luc CCMF PLGA NPs). (**f**) Metastatic burden quantification in lungs determined from the percentage of metastatic nodule area to the total lung area. * *p* < 0.05 (n = 5). Figure adapted with permission from *ACS Appl. Mater. Interfaces* **2019**, *11*, 7850–7861 (https://pubs.acs.org/doi/10.1021/acsami.8b22309). Copyright 2019 American Chemical Society (accessed on 1 March 2022).

Tumor cell coated NPs can be used to interfere with the tumor reprogramming of parenchymal cells, acting as antagonists to cell–cell interactions. A study by Jin et al. [59] demonstrated how U87 tumor cell membrane coated PLGA NPs (U87-CXCR4-CCMF-PLGA-NPs) had the dual action of reducing tumor cells interactions with fibroblasts, and at the same time providing tumor neoantigens to induce an antitumor immune response (Figure 7a). These activities were also present in vivo, with reduced liver and increased lung accumulation (Figure 7b), despite shorter circulation (Figure 7c), and reduced the number and size of metastases by over 90% (Figure 7e,f) in models of metastatic breast cancer models. They also accumulated in lymph nodes, presenting their antigen to immune cells, and thus stimulating CD4 and CD8 T cells, which could result in antitumor immune response.

Tumor cell-coated NPs for tumor vaccines can also be modulated using genetic engineering to alter their expression of surface markers [60]. A recent work by Jiang et al. [61] focused on the use of B16 melanoma cells as a starting material to create membrane coated PLGA NPs for antigen presentation with the aim to induce an antitumor immune response. The source tumor cells were transfected to induce the expression of the co-stimulatory receptor CD80 and an ovalbumin antigen (OVA) as adjuvants to improve immune response. These membrane-coated particles demonstrated high CD80 presentation, remarkable stability, and efficiently induced the expression of several inflammatory markers by splenocytes in vitro, over doubling the amount of memory cells compared to all other combinations of CD80 and OVA, and more than 80-fold increase in the secretion of pro-inflammatory cytokines such as IL-2 and IFN-γ. After intravenous injection, these NPs prevented tumor growth when used before tumor cells engraftment and to retard tumor growth in already tumor baring mice, confirming their accumulation in lymph nodes where they elicited T-cells stimulation and resulted in improved animals’ survival.

Another example of tumor nanovaccines is given by Kroll et al. [62], who loaded PLGA NPs with the adjuvant CpG oligodeoxynucleotide 1826 to induce the maturation of dendritic cells (DCs) and coated them with melanoma cells membrane to present multiple antigens, aiming to elicit an antitumor immune multiantigen-based response. This platform induced DCs maturation more efficiently than tumor cell membranes alone, doubling the expression of DC markers such as CD40, CD80, and CD86, as well as doubling CD8+ T-cells proliferation. The nanovaccines elicited antitumor immunity both as prophylactic treatment and as treatment post-challenge in a melanoma murine model, working in synergy with immune checkpoint inhibitors and largely improving animals’ survival.

### 2.5. Multiple Membrane-Coated NPs

It is possible to combine membranes from different cells onto a single nanovector surface. This would result in NPs with the advantages of all the cell membranes they are coated with. This hybrid-based strategy offers another level of complexity to biomimetic NPs.

A proof of concept of this intuition is offered by Gong et al. [63]. In this work, DOXO loaded PLGA particles were coated with a mix of RAW264.7 murine macrophages and breast cancer 4T1 cells. The fusion of these membranes onto the surface of single particles was confirmed by Fluorescence Resonance Energy Transfer (FRET) analysis. The hybrid system demonstrated improved tumor cells targeting in vitro compared to either the macrophage or tumor cells coated NPs as well as improved tumor cell killing capacities. Furthermore, this new system demonstrated improved tumor accumulation in vivo (30% more than its respective control), 50% less liver accumulation, and almost 90% reduction of metastatic foci in murine models of metastatic breast cancer, more than doubling animals’ survival.

An exciting frontier in nanotechnology and nanomedicine is offered by nanorobots [64]. These constructs can perform complex behavior, such as external or fuel-induced movement, making them suitable for precision medicine. A recent work from Esteban-Fernández de Ávila et al. [65] aimed to improve nanorobots biocompatibility by coating gold nanowires with a hybrid PT and RBC membrane to neutralize Multidrug Resistant *Staphylococcus aureus* (MRSA) and pore forming toxins. These metal NPs can propel themselves in solution when exposed to ultrasounds, making them suitable as mobile nanosponges to capture pathogens very quickly. The coated nanorobots demonstrated improved movement in vitro in whole blood compared to bare nanowires, and at the same time removed very efficiently from the blood pore forming toxins and MRSA.

### 2.6. NPs Coated with Other Membranes

Another approach of the biomimetic strategy is the use of pathogen-coated NPs that can prevent the pathogen adhesion to the target tissue [66], acting as a functional antagonist. This possibility was demonstrated by Zhang et al. [67], who formulated *Helicobacter pylori* membrane coated PLGA NPs. These particles adhered to the stomach epithelium and when the tissue was pretreated in an ex vivo model, they inhibited by almost six times bacterial adhesion. The same group tested also a complementary approach by coating PLGA NPs with the membrane of gastric epithelial cells [68]. These NPs maintained the membrane markers of their source cells and efficiently adhered to *H. pylori* colonies, and when loaded with the antibiotic clarithromycin in the polymeric core, demonstrated a steady release and a synergistic effect with improved antibacterial efficacy in vitro and in vivo reducing more than 50% bacteria proliferation compared to bare NPs.

In another work by Yang et al. [69], pseudoviral antigens were coated with yeast-derived polyethyleneimine membranes to formulate orally active vaccines. The yeast wall offers protection from gastric degradation for the protein antigens, and it also enable active targeting of local intestinal immune cells. These vectors were injected in the intestinal lumen of healthy mice and demonstrated to be well tolerated and efficiently accumulate in the Peyer’s patches, which are the main foci of immune activity within the intestine. This system could enable the development of simple vaccines for many other pathologies in the future.

## 3. Cell Membrane and Membrane Derived Nanovesicles

### 3.1. Membrane Nanovesicles

Among the different biomimetic nanoplatforms, there is also the possibility to use the membranes closed onto themselves to generate nanovesicles as standalone nanovectors. This strategy was followed by Oieni et al. [70], who generated cell-derived nanoghosts (NGs) from many different cell lines. This technology employs a series of hypotonic treatment of cells, sequential centrifugation, and sonication to remove most of the cellular organelles and isolate the membranes. This technique is fast and easy to perform, and can be applied to many different cells, yielding high amounts nanovesicles. It is possible to perform drug loading or labelling of nano ghosts either by treating the source cells with the desired drugs, from which the cargo is maintained into NGs, or by post-loading, loading or labeling NGs during or after their production though extrusion or sonication. NGs were thus successfully loaded with fluorescent labels, radioisotopes, and both small-molecules drugs as well as DNA [71].

NGs were applied to MSCs transfection with an anti-miRNA-221 antisense oligonucleotide by loading via electroporation [72]. miRNA-221 is considered an important novel player in bone disease development [73]. In this work, NGs were efficiently endocytosed by MSCs, accumulated in the endosomes and efficiently the oligonucleotide efficiently from the endo-lysosomal compartment. NGs demonstrated comparable miRNA-221 knockdown efficiency to the more cytotoxic cationic lipofectamine. Furthermore, these NGs efficiently delivered their cargo to MSCs in a murine e osteochondral defect model.

A similar study from Kaneti et al. [74] demonstrated that MSCs-derived NGs can be loaded with plasmids. Specifically, the authors encapsulated a plasmid encoding for hemopexin-like domain, an onco-suppressive protein to both tumor cells and tumor vasculature. This nanovector induced tumor cells death and inhibited endothelial cells proliferation and migration by 50%, increasing the expression of the hemopexin in the target cells. This translated to a 70% reduction in tumor growth in in vivo models of prostate cancer and significantly reduced the amount of tumor foci in a murine model of lung cancer. This system provided reliable results in very different pathological settings, demonstrating its versatility and high therapeutic potential.

Another interesting membrane vesicle strategy has been investigated by Han et al. [75], who fused tumor cells and RBCs membranes to obtain nano-antigen-erythrosomes (Ag-erythrosomes). This system exploits as the natural tropism of RBC fragments towards the spleen, where senescent RBCs are physiologically cleared. The injection of these NPs resulted in their efficient accumulation in mice spleen, and the subsequent increase in increased presence of splenic NK cells, T cells, B cells, as well as increased levels of pro inflammatory IL-6, TNF-α, and IFN-α/γ in the serum. The intravenous injection of Ag-erythrosomes in melanoma bearing mice resulted in the local infiltration of CD4+ and CD8+ T cells, demonstrating a synergistic effect in combination with antiPD-L1 antibodies as immune checkpoint inhibitors. This ultimately led to a much-slowed tumor growth rate. Ag-erythrosomes also efficiently reduced tumor recurrence and metastasis formation in vivo after surgical resection of the primary tumor mass. This study underlines how exploiting physiological immune clearing mechanisms can lead to innovative targeting strategies towards immune cells to achieve in vivo immunotherapy.

A similar approach was recently offered by Zou et al. [76], who fused bacterial membrane vesicles with tumor cells membranes to obtain vesicles that can present tumor neoantigens to the immune system, using as adjuvant the intrinsic immunogenic activity of bacterial molecules. When exposed to DCs in vitro, these particles induced their proliferation. In turn these cells could activate CD8+ T cells which exerted a tumor-specific cytotoxic activity on tumor cells that were used for membrane derivation. After intraplantar injection in mice, these composite particles were retained into the local lymph nodes more efficiently than vesicles made solely of tumor cell membranes. These nanovectors demonstrated the ability to induce distal immune response against distal tumor metastasis in vivo and demonstrated the induction of a CD8+ T cells-based immune response in a murine model of TNBC, reducing tumor weight by 60% with no obvious systemic toxicity to the animals.

### 3.2. Membrane Proteins-Based Nanovectors

Another viable approach in biomimetic nanomaterials design is focused on the isolation of membrane proteins and their engraftment on artificial phospholipids bilayers. This strategy allows the removal of intracellular proteins and yields high amounts of materials from virtually any cells.

**Figure 8:**
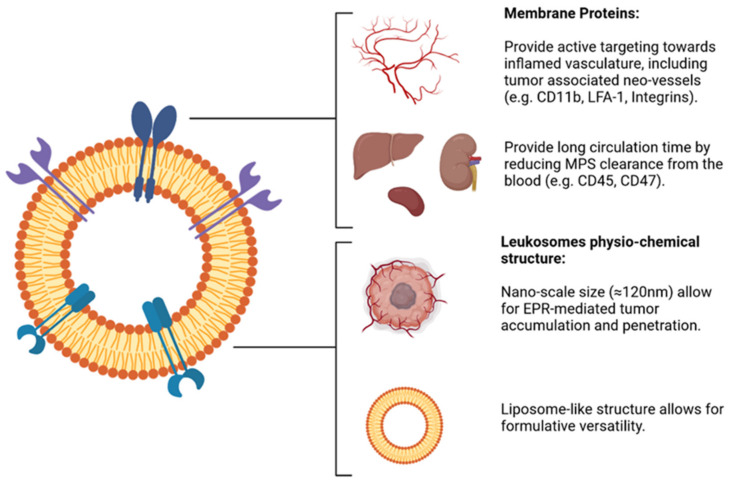
Schematic representation of the structure and functions of leukosomes. EPR: enhanced permeability and retention effect. Image adapted from [77] under creative commons authorization.

In recent years, this intuition was successfully explored by Tasciotti, Molinaro, and Taraballi, creating Leukosomes (Leukos) [77,78]. Leukos are phospholipid and cholesterol-based nanovesicles functionalized with monocytes-derived membrane proteins (Figure 8). The formulation of Leukos is a good example of the complementary use of a “top-down” approach, in which the membrane proteins are purified from leukocytes, and “bottom up” strategy, in which these membrane proteins are integrated in the phospholipid and cholesterol bilayer of Leukos.

Membrane proteins so far have been extracted using a commercial kit used for mammalian cells fractionation, and it is based on the use of different detergent buffers and centrifugation steps to separate the plasma membranes from the other cell organelles, and then to remove membrane proteins from the plasma membrane itself. The use of detergents is essential to remove the native membrane lipids while providing a partially hydrophobic environments within their micelles which cover the natural hydrophobic domains that are present in the integral membrane protein structure. The resulting proteins are kept in their native state while being suspended in an aqueous buffer.

The subsequent integration of membrane proteins into Leukos has so far been performed through two main approaches. The first one is based on the traditional thin layer hydration technique which is also used for Liposomes. Briefly, phospholipids and cholesterol dissolved in chloroform and dried to form a thin lipid layer which is then hydrated with an aqueous solution containing membrane proteins. This hydration induced the replacement of the detergents surrounding the proteins with the lipids, forming coarse and multilamellar vesicles. These large vesicles are then extruded through increasingly small membranes to induce the formation of homogeneous and unilamellar Leukos. Another more recent approach is based on the microfluidics platform NanoAssemblr™. This instrument allows the fast and efficient mixing of two liquid miscible phases to induce the self-assembly of lipid and polymer-based nanoparticles. In the case of Leukos, lipids and cholesterol have been dissolved in ethanol, while the solubilized membrane proteins are dispersed in an aqueous solution. The fast mixing of these two phases causes a sudden change in the polarity of the solvent, inducing the self-assembly of lipids and proteins in nanovesicles in a single step. This approach allows for the fast and scalable formulations of Leukos.

Leukos have been loaded with many drugs, including DOXO, Ponatinib, and paclitaxel, and has been applied to several pathologies as osteosarcoma [79], and breast cancer, demonstrating their remarkable versatility as drug delivery systems, targeting acute inflammation caused by infections or tissue damage, and the intrinsic inflammation characterizing the stroma of many tumor. In all these cases, Leukos was demonstrated to be highly biocompatible and improved targeting efficiency and therapeutic efficacy compared to the respective drug. The successful engraftment of leukocytes membrane proteins on the particles surfaces was verified by western blot and flow cytometry [80] to confirm the correct orientation of membrane proteins onto the particles surfaces.

Leukos also demonstrated their therapeutic potential as stand-alone medical devices. Indeed, drug-free Leukos were used to treat sepsis [81], a systemic inflammatory condition caused by circulating bacteria. Leukosomes interacted with circulating monocytes in murine sepsis models and modulated the expression of key genes in macrophages such as IL-1β, IL-6, IL-10, TNF-α, and TGF-β towards an anti-inflammatory profile. This effect was produced on inflamed macrophages but not on inflamed endothelial cells. This immune modulation resulted in an overall decreased sepsis score by four times in mice and increased survival.

A similar drug-free application of Leukos is their application in inflammatory bowel disease (IBD). In this case, intravenously administered Leukos accumulated into the intestinal tissue of via endothelial adhesion and exerted intrinsic anti-inflammatory activity in murine models of IBD [82]. This study investigated the behavior of specialized Leukos obtained from macrophages that were stimulated with retinoic acid to induce the overexpression of integrins, formulating specialized Leukos (SLKs) with improved tissue targeting and therapeutic effect compared to traditional liposomes. SLKs reduced the immune cells by 60% and TNF-α by over 90% in the inflamed tissue, resulting in 50% reduced pathological score and reduction of intestinal lesions. The mechanisms of action of Leukos and SLKs could derive from their ability to occupy the adhesion molecules expressed by inflamed endothelia, preventing further accumulation of immune cells in the intestine, and allowing its recovery. It is also possible that Leukos act as a molecular sponge, absorbing proinflammatory cytokines via their surface receptors. Nevertheless, this study provides the possibility to modulate the surface proteins profile of cells used as starting material for Leuko production.

### 3.3. Techniques Used for Membrane Coating or Membrane/Based Nanovectors Assembly

Table 1 summarizes the advantages and disadvantages of each cellular source discussed in the previous sections of the article, as well as the main techniques used to purify cell membranes and their components and integrate them in biomimetic nanovectors.

Despite slight differences based on the specific cell line in study, most membrane extraction protocols are based on a first step of cell isolation, usually from cell cultures or whole blood, performed through centrifugation. This also helps to remove all the unwanted components of cell culture medium or plasma. Cells are then disrupted mechanically or chemically to separate the membranes (i.e., hypotonic treatment, sonication, homogenization) or membrane proteins (i.e., detergent-containing buffers from the other cell organelles.

Membranes are finally purified by differential or gradient centrifugation. In the case of membrane proteins, the solubilized macromolecules are retained in the supernatant after centrifugation to remove the residual cellular fragments.

Further sonication is employed to obtain membrane vesicles that can be used as a standalone nanovectors. Conversely, NPs coating is normally performed by multiple extrusion steps, sonication, and in some instances electroporation.

Thus, the techniques used for membrane coating or membrane assembly into nanovectors appear to be uniform and well established. However, further optimization in terms of scalability, routine quality control for membrane purity, and establishment of ideal long terms storage conditions to retain membrane integrity and function are all critical issues that warrant further research.

## 4. Current Hurdles and Future Perspectives on the Development of Biomimetic NPs

All the approaches for the formulation of biomimetic NPs discussed above can achieve membrane reconstitution onto nanoparticles. However, all of them present some limitations that are worth considering.

A general caveat for the use of biomimetic nanomaterials is a fruit of their own complexity. Specifically, it is quite difficult to understand the relevance of specific molecules in defining the nanomaterial behavior, since the entire membrane could present hundreds of different molecules. So far, only few studies have attempted to specifically block single membrane proteins using antibodies to study how the particle behavior changed without their function. However, it is possible that more proteins could redundantly have the same functions, or that some unexpected protein provide important contribution to the behavior of formulations. Thus, the investigation of this new level of complexity could require in the future more efforts with innovative techniques that offer a more in depth understanding. Expanding the knowledge and defining the essential molecules required for the desired particle function could ultimately lead to the production of nanomaterials in which single proteins that are found in disparate cell types are combined on a single platform, bypassing the necessity to bring along a lot of unwanted cellular material. This could result in simpler formulations with more reproducible features and easier scalability. Furthermore, despite the surface proteins having gathered the most attention from the research so far, the biological function of the cells’ membrane can be provided by the phospholipid bilayer they are inserted in, and by the complex sugars onto the proteins and lipids. To the best of our knowledge, there are almost no studies on these specific components of the membranes, and further investigation could provide more options for innovative biomimetic nanosystems.

Another important issue has recently been raised in a study by Liu et al. [83] Specifically, the authors demonstrated how extrusion, sonication, and the common ratios between particles and membranes used for NP coating result in formulations with a very small percentage of completely coated particles. Of note, the authors proved that partially coated NPs were internalized by cells via different mechanism compared to completely coated cells. This discovery is of critical importance to improve the current formulative approaches biomimetic membrane coated nanovectors. Indeed, an optimized NPs coverage with cell membranes could result in improved colloidal stability, prolonged pharmacokinetics, and maximized efficacy thanks to the increased biological payload carried by the nanoparticles. Most studies so far relied on qualitative techniques for the assessment of NPs coverage, including TEM imaging, zeta potential assessment, or SDS-PAGE to confirm the transposition of membrane proteins. Thus, quantitative assessment of NP membrane coverage is warranted in future formulations.

A final hurdle to address is the regulatory classification of biomimetic nanomaterials. The biological source of biomimetic materials not only requires overcoming the regulatory limitations of traditional nanomaterials, but their complexity requires to consider ad hoc validation guidelines for their production and safety. Indeed, if membrane-based biomimetic technology aims to become the new frontier in the development of nanovectors for biomedical applications, it is necessary to gain a deeper understanding of the interactions between the membrane components and the biological environment biomimetic materials meet after injection. This would lead to the construction of a biomimetic materials toolbox that would allow the formulation of membrane coated or membrane-based nanovectors with highly optimized design to achieve specific behaviors in a multitude of theranostic applications.

## Figures and Tables

**Table 1:** Summary of the different advantages and disadvantages for each cell line used for biomimetics formulation, as well as the techniques used for membrane and membrane components isolation and integration in nanomaterials.

Starting Cells	Advantages	Disadvantages	Technology Used for Biological Material Derivation	Technology Used to Integrate the Membranes on Nanovectors or Induce Vesicles Formation	References
Red blood cells	-Abundant in the blood and easy to separate. -Ease of membrane isolation ^1^ -Provide longer circulation. -Provide decoy against RBCs targeting toxins.	-No intrinsic targeting moieties -Keep in mind the blood type used -HEME group residues potentially toxic	-Whole membranes: whole blood centrifugation to remove plasma, platelets, and other cells; hypotonic treatment to lyse isolated RBCs, followed by centrifugation to remove hemoglobin.	-Membrane coating: mixing with synthetic particles followed by sonication and/or extrusion to reduce size and make the particles homogeneous in size. -Vesicle formation: sonication and/or extrusion to reduce size and make the particles homogeneous in size.	Membrane coating: [32,33,34,35,36,37,38,39,40,41,42,43,44,45,46,65] Nanovesicles formulation: [75]
Platelets	-Relatively easy to separate. -Ease of membrane isolation ^1^ -Provide longer circulation. -Targeting to inflamed/damaged tissue	-Less abundant in the blood.	-Whole membranes: whole blood low speed centrifugation to remove red and white blood cells; higher speed centrifugation to wash away the plasma; followed by freeze and thaw cycles to separate the platelets membranes.	-Membrane coating: mixing with synthetic particles followed by sonication and/or extrusion to reduce size and make the particles homogeneous in size.	[47,48,49,50,51,52,65]
Leukocytes	-Active targeting to inflamed tissue. -Wide variety of different cell populations. -Possible manipulation of membrane proteins.	-Not very abundant in the blood. -Requires removal of all non-membrane cellular contents. -Effect on plasmatic half-life variable.	-Whole membranes: homogenization, followed by sequential centrifugation to remove cell organelles. -Membrane proteins extraction: detergent-based cell permeabilization to remove intracellular components followed by plasma membrane solubilization using stronger, non denaturating detergents	-Membrane coating: mixing with synthetic particles followed by sonication and/or extrusion to reduce size and make the particles homogeneous in size. -Biomimetic nanovesicles formulations (leukosomes): membrane proteins integration through lipids hydration with membrane proteins suspension and subsequent extrusion; or single step integration of membrane proteins into the forming vesicles through microfluidics.	Membrane coating: [53,54,55,56,57,58] Leukosomes formulation: [77,78,79,80,81,82]
Cancer Cells	-Possible homologous targeting to tumor cells. -Tumor neo-antigens for nanovaccines.	-Difficult to obtain enough autologous, patient-specific cells. -Requires removal of all non-membrane cellular contents. -Possible safety concerns.	-Whole membranes: homogenization, followed by sequential centrifugation to remove cell organelles. In some cases, membranes are further purified using gradient centrifugation.	-Membrane coating: mixing with synthetic particles followed by sonication and/or extrusion to reduce size and make the particles homogeneous in size.	[59,60,61,62,63]
Bacterial cells	-Allow the display of bacterial antigens without risk of active infection -Antagonize bacterial adhesion to tissues	-Highly antigenic material	-Centrifugation of confluent bacterial cultures to separate bacteria from spontaneously released membrane vesicles and subsequent filtration to ensure the absence of living bacteria.		[67]
Other mammalian cells	-Highly dependent on the cell type, but often based on the translation of specific receptor and binding proteins onto nanomaterials	-Requires removal of all non-membrane cellular contents. -Possible difficulties in having large amounts of starting materials	-Whole membranes: homogenization, followed by sequential centrifugation to remove cell organelles.		[68]
Mesenchymal stem cells	-Provide active targeting to solid tumors.	-Requires removal of all non-membrane cellular contents. -Complex isolation or stabilized cell lines.	-Whole membranes: hypotonic lysis, homogenization with a dunce homogenizer, and differential centrifugation to separate membranes from cell debris.	-Vesicles formation (Nanoghosts): sonication and/or extrusion to reduce size and make the particles homogeneous in size.	[72,74]

^1^ No nuclei and few or no intracellular organelles.

## Data Availability

Not applicable.
